# Ethanol Measurement Using Hetero-Core Structured Optical Fiber Covered with Layer-By-Layer Thin Film

**DOI:** 10.3390/foods7080117

**Published:** 2018-07-25

**Authors:** Yuko Kimura, Atsushi Seki, Kazuhiro Watanabe

**Affiliations:** Department of Science and Engineering for Sustainable Innovation, Faculty of Science and Engineering, SOKA University, Hachioji, Tokyo 192-8577, Japan; kimuvida36@gmail.com (Y.K.); kazuhiro@soka.ac.jp (K.W.)

**Keywords:** hetero-core structured optical fiber, layer-by-layer thin film, ethanol sensor

## Abstract

Ethanol measurements are performed in an ethanol/water solution utilizing an ethanol sensor based on a hetero-core structured optical fiber covered with a layer-by-layer thin film. The layer-by-layer (LbL) thin film was prepared using poly (allylamine hydrochloride) and poly styrene sulfonate. When the sensor was immersed in water, the propagating light intensity decreased with increasing ethanol concentration. This behavior suggested that the LbL film contracted due to the presence of ethanol, and the refractive index of the film increased, resulting in increasing propagating light leaks at the hetero-core of the fiber. The ethanol sensor was applied to a variety of spirits, and the propagating light intensity decreased with increasing ethanol concentration.

## 1. Introduction

Optical fibers have been used as telecommunication media because of their considerable advantages, such as their small size, lightweight construction, flexibility, immunity to electromagnetic interference, and low cost. Due to their chemical stability, optical fibers have also recently attracted attention as chemical sensing elements [1]. 

Optical fibers have a core surrounded by cladding that confines the propagating light to the core; thus, propagating light is not able to interact with the outer environment in intrinsic mode [2]. In order to be used as chemical sensors, the propagating light must be able to interact with the outside environment. Thus, many types of optical fibers have been developed that enable interaction between the outside environment and core, such as core-exposed fibers and tapered fiber optics, and many kinds of chemical sensors and biosensors have been based on these fiber optics [3,4,5]. 

Ethanol measurement is important for many fields such as clinical analysis, quality control in food and beverages, and fermentation control. Over the years, many types of ethanol sensors have been reported [6]. However, ethanol is a flammable and volatile material. Unlike many of the previously proposed sensors, fiber optic sensors do not have electrical contacts in the sensing component. Therefore, one advantage to the use of fiber optic ethanol sensors is reduced risk of fire.

Many types of fiber optic ethanol sensors have been reported. In these sensors, surface plasmon resonance (SPR) and/or localized surface plasmon resonance (LSPR) was used to measure the ethanol concentration based on the refractive index change [7,8,9]. 

**Figure 1:**
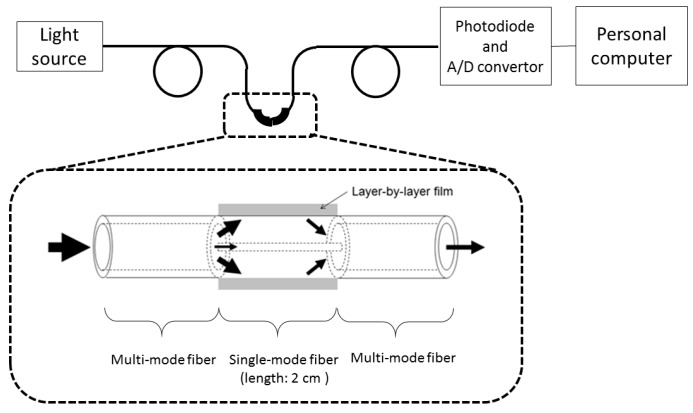
Schematic diagram of the measurement system and sensor structure.

As a novel structure in optical fiber sensors, a hetero-core structured optical fiber was developed and has been applied to chemical sensing [9,10,11,12]. The hetero-core structured optical fiber consists of short piece of single-mode optical fiber that functions as a sensing part and multi-mode optical fibers that function as the transmission line (Figure 1). The fabrication of hetero-core structured optical fiber is easy because it is fabricated using a commercially available fusion-splicer. In addition, the mechanical strength is enough because the cladding of the sensing part is not reduced.

In this primary study for the application of a hetero-core structured optical fiber to the food field, a simple fiber optic structure is reported based on Snell’s law, in which expansion or contraction of the layer-by-layer (LbL) thin film in the presence of ethanol results in a change of the refractive index of the film.

## 2. Materials and Methods

### 2.1. Materials

Poly (allylamine hydrochloride) (pAA) was purchased from Sigma-Aldrich Corp, Missouri, USA, poly (sodium p-styrenesulfonate) (pSS) was purchased from Acros Organics, New Jersey, USA, and 3-aminopropyl trimethoxysilane was purchased from Tokyo Chemical, Tokyo, Japan. The alcoholic beverages used as samples included non-alcoholic beer, beer (ethanol concentration is 5%), white wine (12%), Kirin-Syotyu (12%), Daigoro-Syotyu (25%), and gin (47%). Other chemicals were analytical grade. A step index (SI)-type single-mode (SM) optical fiber (cladding diameter of 125 µm and core diameter of 3 µm) was purchased from Newport Corp, California, USA. A graded-index (GI)-type multi-mode (MM) optical fiber (cladding diameter of 125 µm and core diameter of 50 µm) with an FC connector was purchased from MIKI Inc., Tokyo, Japan. 

### 2.2. Sensor Fabrication and Measurement System

The fabrication of the hetero-core structured optical fiber, the measurement system, and the deposition of the layer-by-layer thin film has been described elsewhere [10,11]. In brief, the surface of the hetero-core structured fiber optic was treated with 3-aminopropyl trimethoxysilane to introduce a positive charge, and the aminated surface was alternatively immersed in a 20-mM pSS solution and a 20-mM pAA solution for 30 s. The propagating light intensity was recorded for monitoring the ethanol concentration and the deposition process. 

In the measurements of the ethanol/water solution and the alcoholic beverages, a hetero-core fiber optic covered with 80 layers of pSS and pAA (pSS/pAA)_80_ was used. The sensor was immersed in the solution, and the propagating light intensity was measured.

## 3. Results and Discussion

Figure 1 shows the schematic diagram of the measurement system and sensor structure. The nearly white light propagating in the core of the MM fiber leaked to the cladding of the SM fiber after passing through the spliced junction because the core diameter of the SM fiber was smaller than that of the MM fiber. Because the refractive index of the sensor surround was larger than that of the cladding, leakage of the cladding light into the surround increased based on Snell’s law. The propagating light intensity decreased with the increasing refractive index of the SM fiber’s cladding material.

**Figure 2:**
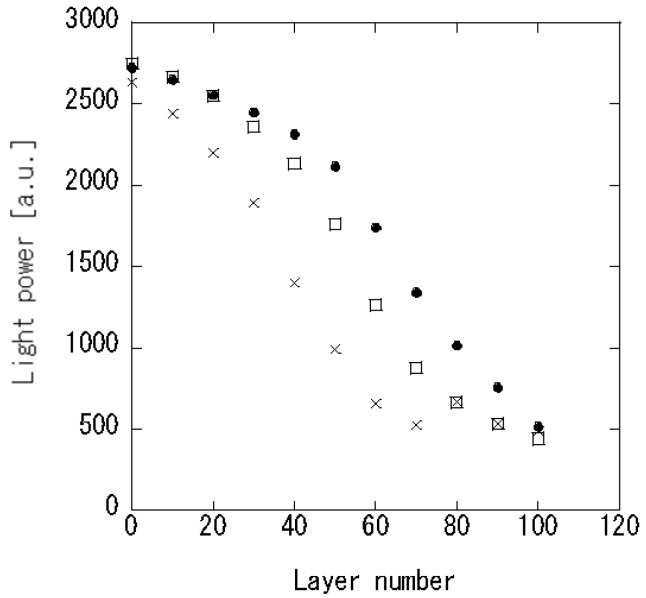
Propagating light intensity of the 60-layers deposited sensor in air (-●-), water (-□-) and ethanol (-×-).

Figure 2 shows the correlation between the propagating light power at 600 nm and deposited layer number. According to measurements taken during the deposition cycle, the propagating light power decreased with increasing LbL film thickness. Deposition of LbL thin film caused an increase of the refractive index in the vicinity of the sensing component. In the sensing cladding, many propagating light modes were generated. This behavior indicates that leakage of the propagating light within the sensor increased according to Snell’s law, resulting in a decrease of the propagating light intensity.

**Figure 3:**
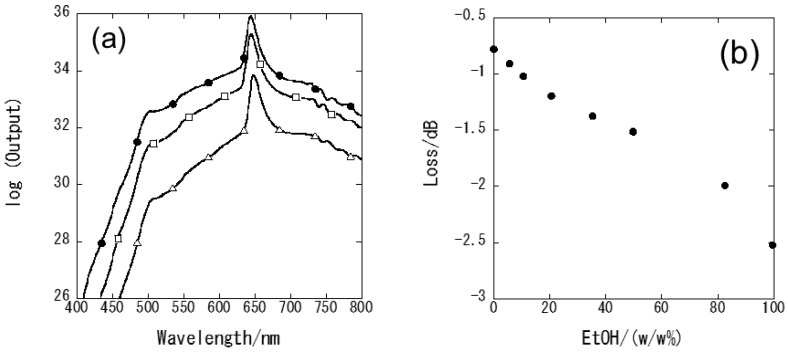
(**a**) Propagating light spectra of the 60-layers deposited sensor in air (-●-), water (-□-) and ethanol (-△-), and (**b**) the correlation between ethanol concentration and propagation loss at 600 nm.

Figure 3a shows the propagating light spectra for the 60 layers-deposited fiber in air, water, and ethanol. The propagating light intensity of the sensor decreased over the wide wavelength range considered with the highest propagation light intensity occurring in air (refractive index of 1.00), followed by that in water (refractive index of 1.33) and ethanol (refractive index of 1.36). Figure 3b shows a correlation between the ethanol concentration and the sensor output for measurements performed in various ethanol/water solutions. The propagating intensity decreased monotonically with increasing ethanol concentration.

**Figure 4:**
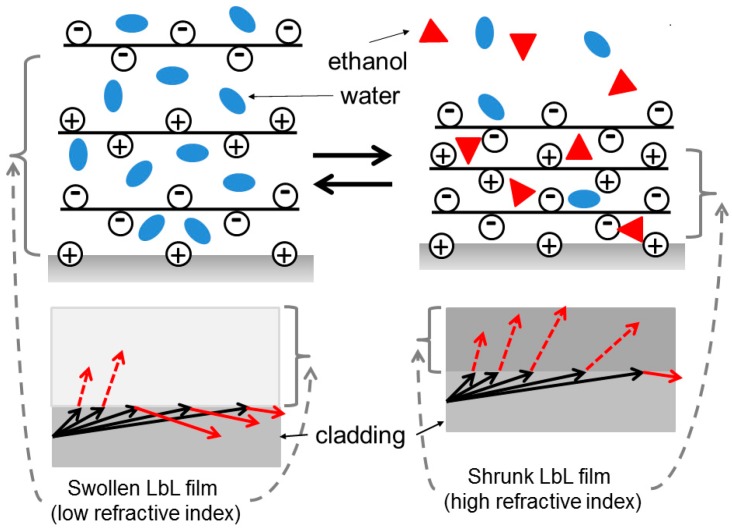
Schematic diagram of the response mechanism of the LbL film to ethanol. (Black solid arrows indicate the propagating light in the cladding. Red solid arrows indicate the reflected light. The dotted red arrows indicate the refracted light.).

In addition, the degree of the decreasing propagating light intensity of the LbL film-deposited fiber was larger than that of the bare fiber. This difference was attributed not only to the refractive index of the solution but also to the contraction of the LbL film in the presence of ethanol [13]. The schematic is presented in Figure 4. 

When the sensor was immersed in water, water (refractive index of 1.333) permeated the LbL film, and any air (refractive index of 1.00) within the LbL film was pushed outside of the film. Likewise, when ethanol (refractive index of 1.36) was added to the water, the refractive index of the LbL film increased. In addition, the LbL film contracted in the presence of ethanol further increased the refractive index, resulting in increased propagating light leakage and decreased propagating light intensity. 

**Figure 5:**
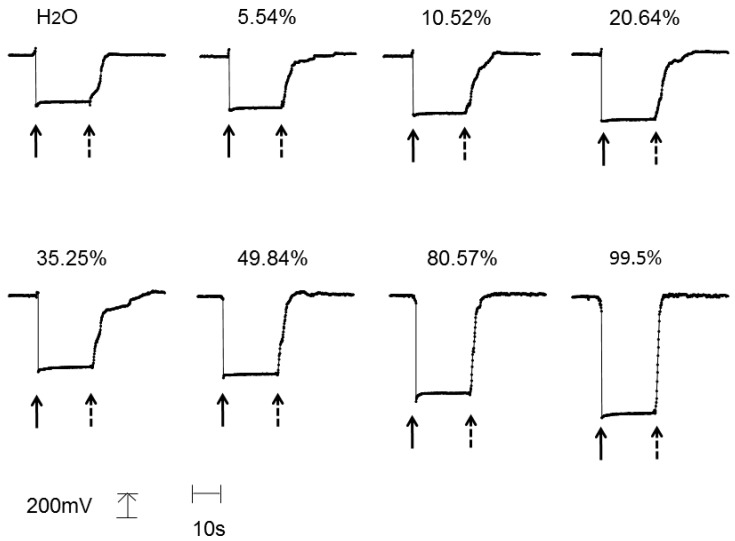
Response curve of the fiber optic ethanol sensor. The vertical scale indicates the sensor output (the length is equivalent to 200 mV), and the horizontal line indicates time (the length is equivalent to 10 s). Solid arrows indicate the dipping the sensor into the solution, and dotted arrows indicate the raising the sensor from the solutions.

Figure 5 shows the response curve of the sensor. The transmitted power at 600 nm was recorded when the sensor was immersed in (solid arrow) and/or pull up from (dotted arrow) the various concentrations of ethanol solution. The sensor responded as soon as it was dipped into the solution. The sensor output increased with increasing ethanol concentration. When the sensor was removed from solutions with 5.54–49.84% ethanol concentration, the sensor output was initially slow to respond. On the other hand, the sensor output showed a rapid response when the sensor was removed from concentrations of 80.57% and 99.5% ethanol. The vapor pressure of ethanol is larger than that of water at the same temperature. Therefore, ethanol evaporates faster than water. A first quick response would be attributed to the evaporation of ethanol from the LbL film, and the following slow response would be attributed to the evaporation of water. 

**Figure 6:**
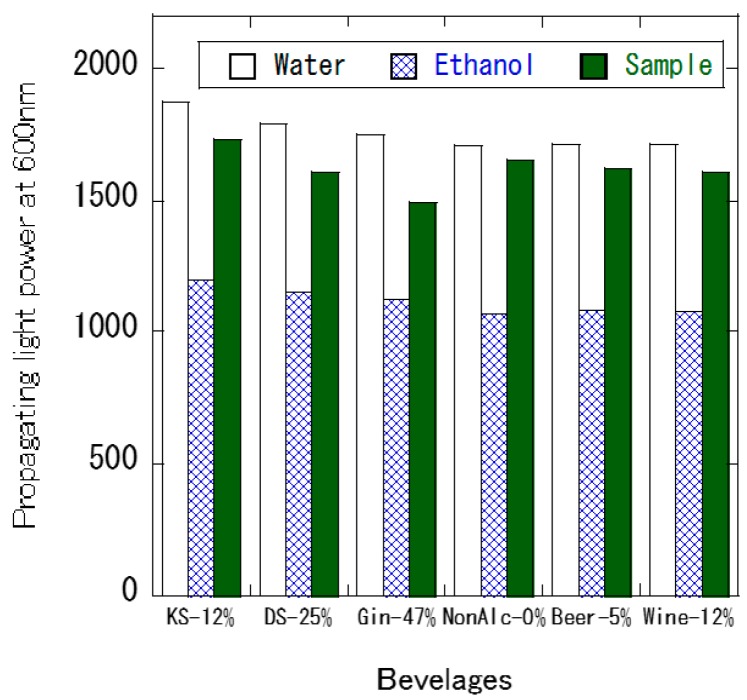
The propagating light intensity within various beverages. The sensor was subsequently immersed in water, ethanol, and the sample. KS–12%: Kirin Shotyu (12% ethanol); DS–25%: Daigoro-Shotyu (25% ethanol); Gin–47%: Gin (47% ethanol); NonAlc–0%: non-alcohol beer (0% ethanol); Beer–5%: beer (5% ethanol); Wine–12%: White wine (12% ethanol).

The sensor was then immersed in alcoholic beverages, and the propagating light intensity was measured. The sensor was subsequently immersed in water, ethanol, and the alcoholic beverage sample. Figure 6 shows the propagating light intensity of the sensor when the sensor is immersed in alcoholic beverages such as non-alcoholic beer (ethanol concentration is 0.00%), beer (5%), white wine (12%), Kirin-Syotyu (12%), Daigoro-Syotyu (25%), and gin (47%). The sensor was tested in this order of the beverages. In distilled alcoholic beverages such as Syotyu and gin, the propagating light intensity decreased with increasing ethanol concentration. Likewise, in non-alcoholic beer and beer, the propagating light intensity was smaller in non-alcoholic beer than in beer, showing decreasing propagating light intensity with increasing ethanol concentration. In beers, dissolved carbon dioxide presents weak acidity. In polyelectrolyte multilayer films, pH change in the solution caused expansion and contraction of the film [14]. Therefore, change in the refractive index of the LbL film might occur by the expansion and/or contraction of the LbL film due to the presence of dissolved carbon dioxide. Thus, measurement of ethanol concentration in beverages might require pretreatment to avoid the presence of interfering substances. In Figure 6, the propagating light intensity in water and ethanol decreased in proceeding measurements. In distilled alcoholic beverages, fragrance components are included, and it is assumed that the refractive index of the film might be attribute to adsorbing these components.

## 4. Conclusions

A hetero-core structured optical fiber covered with LbL film was applied to measure ethanol concentration. The propagating light intensity responded to the ethanol concentration in the ethanol/water solution. The mechanism of the response was based on swelling or shrinking of the LbL film, resulting in a refractive index change of the LbL film. The sensor responded to a wide range of ethanol concentrations in water. In applications to real samples, however, pH changes interfered with the sensor output. In addition, it was assumed that the film responded to flavor compounds, and it is necessary to protect the film from interference substances that are adsorbed to the film and change the refractive index of the film.
